# Natural Bacterial Assemblages in Arabidopsis thaliana Tissues Become More Distinguishable and Diverse during Host Development

**DOI:** 10.1128/mBio.02723-20

**Published:** 2021-01-19

**Authors:** Kathleen Beilsmith, Matthew Perisin, Joy Bergelson

**Affiliations:** a Department of Ecology and Evolution, University of Chicago, Chicago, Illinois, USA; University of California, Irvine

**Keywords:** 16S RNA, environmental microbiology, microbial communities, plant-microbe interactions

## Abstract

To study the spatial and temporal dynamics of bacterial colonization under field conditions, we planted and sampled Arabidopsis thaliana during 2 years at two Michigan sites and surveyed colonists by sequencing 16S rRNA gene amplicons. Mosaic and dynamic assemblages revealed the plant as a patchwork of tissue habitats that differentiated with age. Although assemblages primarily varied between roots and shoots, amplicon sequence variants (ASVs) also differentiated phyllosphere tissues. Increasing assemblage diversity indicated that variants dispersed more widely over time, decreasing the importance of stochastic variation in early colonization relative to tissue differences. As tissues underwent developmental transitions, the root and phyllosphere assemblages became more distinct. This pattern was driven by common variants rather than those restricted to a particular tissue or transiently present at one developmental stage. Patterns also depended critically on fine phylogenetic resolution: when ASVs were grouped at coarse taxonomic levels, their associations with host tissue and age weakened. Thus, the observed spatial and temporal variation in colonization depended upon bacterial traits that were not broadly shared at the family level. Some colonists were consistently more successful at entering specific tissues, as evidenced by their repeatable spatial prevalence distributions across sites and years. However, these variants did not overtake plant assemblages, which instead became more even over time. Together, these results suggested that the increasing effect of tissue type was related to colonization bottlenecks for specific ASVs rather than to their ability to dominate other colonists once established.

## Abstract (precis)

Developing synthetic microbial communities that can increase plant yield or deter pathogens requires basic research on several fronts, including the efficiency with which microbes colonize plant tissues, how plant genes shape the microbiome, and the microbe-microbe interactions involved in community assembly. Findings on each of these fronts depend upon the spatial and temporal scales at which plant microbiomes are surveyed.

## INTRODUCTION

As plant tissues emerge and grow, new habitats are created for microbial colonists (1, 2). While they represent only a fraction of colonist diversity (3, 4), bacterial endophytes are important to host plant fitness because of their potential to affect nutrient uptake (5–7), stress responses (8), and defenses against pathogens (9, 10). Given these activities, natural and engineered bacterial communities have been proposed as tools for sustainably enhancing plant growth and stress resistance (11). When lineages in these communities are pathogenic or beneficial to the host, the efficiency with which they enter plant tissue is of particular interest (12–14). Despite stochasticity in colonization (15–17), there is evidence that plants selectively filter the bacteria colonizing the intercellular space in their tissues. Characterizing spatial and temporal variation in this filtering is key to understanding how natural and cultivated communities assemble in the endosphere.

The idea that plant tissue filters bacterial colonists is supported by the observation that endophytic communities display only a fraction of the diversity found in soil. For example, the diversity of taxa found in the root endosphere is lower than in rhizosphere soil (18). Furthermore, a subset of bacterial families is found at higher relative abundance in roots than in soil (19). Filtering is likely due in part to differences between soil and root cell walls as substrates for colonization, as indicated by similarities between communities in live roots and wood slivers exposed to the same field-collected soil inocula (20). Filtering by living tissue may also involve selection for or against specific bacterial lineages, as indicated by community members enriched over soil levels in roots but not in wood sliver samples and vice versa (20).

Although plant tissue appears to filter bacterial colonists, we do not yet fully understand how spatial and temporal variation influences this process in natural environments. Variation in abiotic factors and the pool of soil colonists at planting sites can influence the efficiency with which bacterial lineages enter plants, leading to associations between geographic location and the composition of bacterial assemblages in plant tissues (21–23). Recent evidence suggests that within plants, communities in different tissues are composed of a common pool of systemic colonists (24, 25). However, individual lineages can display differences in colonization efficiency between roots and stems (26), and several studies have reported variation between the bacterial assemblages found in different tissues (27–29), supporting the idea that some bacteria are more successful than others in colonizing a given habitat within the plant. Variation in assemblage composition is also observed when plant tissues are sampled at different developmental stages (22, 30, 31). These temporal trends could be related to the time available for bacterial colonization of plant tissues before sampling, changes in how hosts filter colonists with age, or interactions arising as more bacteria cooperate or compete within the host. Since bacteria alter plant tissues upon arrival, the host response to established colonizers could also change the efficacy of colonization later in development (32, 33).

Since most surveys of plant colonists have either focused on a single tissue or taken only a snapshot of community composition in time, it is difficult to compare the extent and interaction of geographic, tissue-level, and temporal effects on plant endophyte filtering. Adding to the body of work exploring host plant control of colonization, we compared the relative influence of plant tissue type, age, harvest site, and year on the bacteria that naturally colonized a common haplotype of an annual plant, Arabidopsis thaliana. To understand the distribution of the bacterial traits driving these patterns, we examined whether relationships between variables and assemblage composition depended on the taxonomic level at which the surveyed colonists were grouped. To ascertain the consistency of spatial and temporal colonization patterns, we compared the tissues and ages at which bacterial lineages reached maximum prevalence or abundance between sites and years. In addition, we characterized how the diversity and evenness of colonists changed across plant tissues throughout development.

## RESULTS

We planted surface-sterilized seeds of *A. thaliana* accessions from a single North American haplotype (see Table S1 in the supplemental material) at two southwest Michigan sites in two consecutive years. These accessions germinate in the fall, overwinter as small rosettes, flower in the spring, and senesce in the early summer. We harvested roots and rosette leaves throughout vegetative growth and also stems, cauline leaves, flowers, and siliques as they became available during flowering and senescence (Table S2). To enrich for endophytes, bacteria were washed from the surfaces of plant tissues by repeated vortexing in surfactant buffer (Text S1). Topsoil was also collected from field sites at each time point during the second year of study. Bacterial lineages in the soil and plant tissue samples were quantified by amplification and sequencing of the V5, V6, and V7 regions of the 16S rRNA gene (16S) (29). The 16S sequences were grouped into amplicon sequence variants (ASVs) with DADA2 (34) in QIIME2 (35). After singleton 16S variants and those from plant organelles had been filtered out, 10,803 ASVs were tallied for 1,272 samples (Table 1). A phylogenetic tree for the variants was inferred with FastTree using MAFFT-aligned 16S sequences (36, 37). The ASVs were classified at seven taxonomic ranks based on the SILVA 16S database (38).

**TABLE 1:** Samples in the study after quality control

Stage of growth	No. of samples (site ME, site WW)							
	Soil	Roots	Rosettes	Stems	Cauline leaves	Flowers	Siliques	Total
No plant	15, 19							34
Two-leaf plant	6, 4	3, 4	8, 10					35
Four-leaf plant		29, 8	27, 29					93
Six-leaf plant	8, 4	32, 18	45, 26					133
Eight-leaf plant		37, 24	41, 35					137
Flowering plant	4, 3	61, 38	64, 49	41, 38	24, 22	42, 28	14, 25	453
Senescent plant	6, 8	60, 64		54, 75			49, 71	387
Total	77	378	334	208	46	70	159	1,272

10.1128/mBio.02723-20.3 TABLE S1 Arabidopsis thaliana ecotypes planted in the study (from the HPG-1 haplogroup). Download Table S1, PDF file, 0.02 MB. Copyright © 2021 Beilsmith et al. 2021 Beilsmith et al. This content is distributed under the terms of the Creative Commons Attribution 4.0 International license.

10.1128/mBio.02723-20.4 TABLE S2 Sample collections for the study. Download Table S2, PDF file, 0.02 MB. Copyright © 2021 Beilsmith et al. 2021 Beilsmith et al. This content is distributed under the terms of the Creative Commons Attribution 4.0 International license.

10.1128/mBio.02723-20.7 TEXT S1 Supplemental methods. Download Text S1, PDF file, 0.09 MB. Copyright © 2021 Beilsmith et al. 2021 Beilsmith et al. This content is distributed under the terms of the Creative Commons Attribution 4.0 International license.

### Bacterial assemblage composition was associated with plant tissue type and developmental stage.

**FIG 1:**
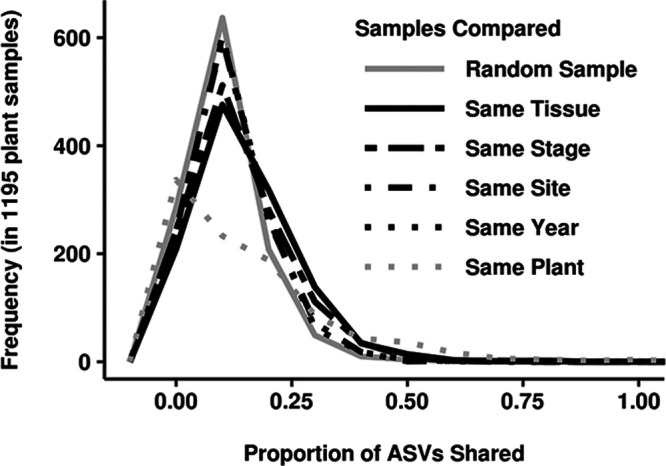
Bacterial assemblage composition was driven by the tissue sampled and the plant's developmental stage at harvest. The frequency distribution for the proportion of ASVs shared by plant samples selected randomly (solid gray line; median, 0.096) differed from those for plant samples selected with respect to tissue type, developmental stage, site, or year (patterned black lines). The proportion of shared members was significantly higher when sample selection was conditioned on each variable, but the strongest shifts were observed for tissue type and developmental stage (same-tissue median, 0.133 [*P* < 2 × 10^−16^]; same-stage median, 0.117 [*P* = 4 × 10^−11^]; same-site median, 0.106 [*P* = 8 × 10^−5^]; same-year median, 0.100 [*P* = 9 × 10^−4^]). The frequency distribution for the proportion of ASVs shared with any samples taken from different tissues of the same individual plant is also shown (dotted gray line; *P* = 0.038).

Plants shaped the bacterial assemblages they hosted, making them distinct from those in the surrounding soil (Text S2). Rather than a single host environment, the plant appeared to be a collection of microbe habitats defined by tissue type and age. Samples from the same tissue or stage clearly shared a higher proportion of members than randomly compared samples (Fig. 1). When samples from multiple tissues of the same individual plant were available, comparisons showed that they did not share a significantly higher proportion of members than randomly paired samples. Bacterial assemblages therefore appeared to be more similar between samples of the same tissue type from different plants than between samples from different tissue habitats in the same plant. Common environments also influenced assemblages, as evidenced by increased membership overlap in samples from the same site or year compared to random samples. Despite these patterns, the low proportion of members shared within groups conditioned on any study variable (<15%) underscored the high variability of colonization.

10.1128/mBio.02723-20.8 TEXT S2 Plants hosted bacterial assemblages distinct from those in the surrounding soil. Download Text S2, PDF file, 0.4 MB. Copyright © 2021 Beilsmith et al. 2021 Beilsmith et al. This content is distributed under the terms of the Creative Commons Attribution 4.0 International license.

The influences of host and environment on assemblage composition were further supported by analysis of variance. After random subsampling to 1,000 counts, composition variation between samples was quantified with respect to ASV presence by Raup-Crick dissimilarity (39), with respect to ASV abundance by Bray-Curtis dissimilarity (40), and with respect to ASV presence and phylogenetic relatedness by the unweighted UniFrac distance (41). Analysis of variance with permutation was performed on each dissimilarity matrix for each study variable (α = 0.001) (Table S3). Variance between ecotypes was not greater than the variance within them, which is unsurprising given the genetic similarity of the haplogroup to which they belonged (42). Variance among plant individuals was significant only when compared by Bray-Curtis dissimilarity. Sample preparation plates and sequencing runs differed significantly in composition only when compared by Bray-Curtis dissimilarity and UniFrac distance. Tissues, developmental stages, planting sites, and years differed significantly in composition regardless of the dissimilarity or distance used to quantify differences.

10.1128/mBio.02723-20.5 TABLE S3 Variables tested for association with dissimilarity matrices by PERMANOVA. Download Table S3, PDF file, 0.03 MB. Copyright © 2021 Beilsmith et al. 2021 Beilsmith et al. This content is distributed under the terms of the Creative Commons Attribution 4.0 International license.

Assemblages were primarily influenced by the type of host tissue sampled and its age. Study variables were nested according to the experimental design to create a multivariate model for permutational analysis of variance (PERMANOVA) (43). When PERMANOVA was performed with this model, tissue type and host developmental stage consistently explained the most sample variation regardless of the dissimilarity metric employed (Table 2). Tissue type was assigned between 13% and 38% of the total variance, and developmental stage was assigned between 6% and 30% of variance. Since the residual sum of squares was markedly lowest in PERMANOVA on the Raup-Crick matrix, we focused on presence-absence variation in community composition when identifying the ASVs associated with specific tissues and developmental stages. An additional advantage of focusing on presence-absence variation is that 16S copy number varies among bacterial lineages.

**TABLE 2:** PERMANOVA results for rarefied data dissimilarity matrix^*a*^

Variable	DOF	Raup-Crick index			Bray-Curtis dissimilarity			UniFrac distance		
		*F*	*R*^2^	Pr (>*F*)	*F*	*R*^2^	Pr (>*F*)	*F*	*R*^2^	Pr (>*F*)
Tissue	17	256.795	0.376	<0.001	7.330	0.127	<0.001	7.949	0.144	<0.001
Stage	7	492.776	0.297	<0.001	12.233	0.087	<0.001	8.048	0.060	<0.001
Site	1	2275.626	0.196	<0.001	46.162	0.047	<0.001	26.216	0.028	<0.001
Year	1	1348.268	0.116	<0.001	28.543	0.029	<0.001	15.496	0.016	<0.001
Sample plate	20	0	0	1	1.346	0.027	<0.001	1.389	0.030	<0.001
MiSeq run	3	0	0	1	3.18	0.01	<0.001	5.59	0.018	<0.001
Residuals			0.015			0.673			0.704	

a Model used for PERMANOVA: Year/Stage/Tissue + MiSeq run/Sample Plate + Site. DOF, degrees of freedom. Pr (>F), probability of F statistic.

### Assemblages in phyllosphere tissues became more distinguishable from those in roots as plants matured.

**FIG 2:**
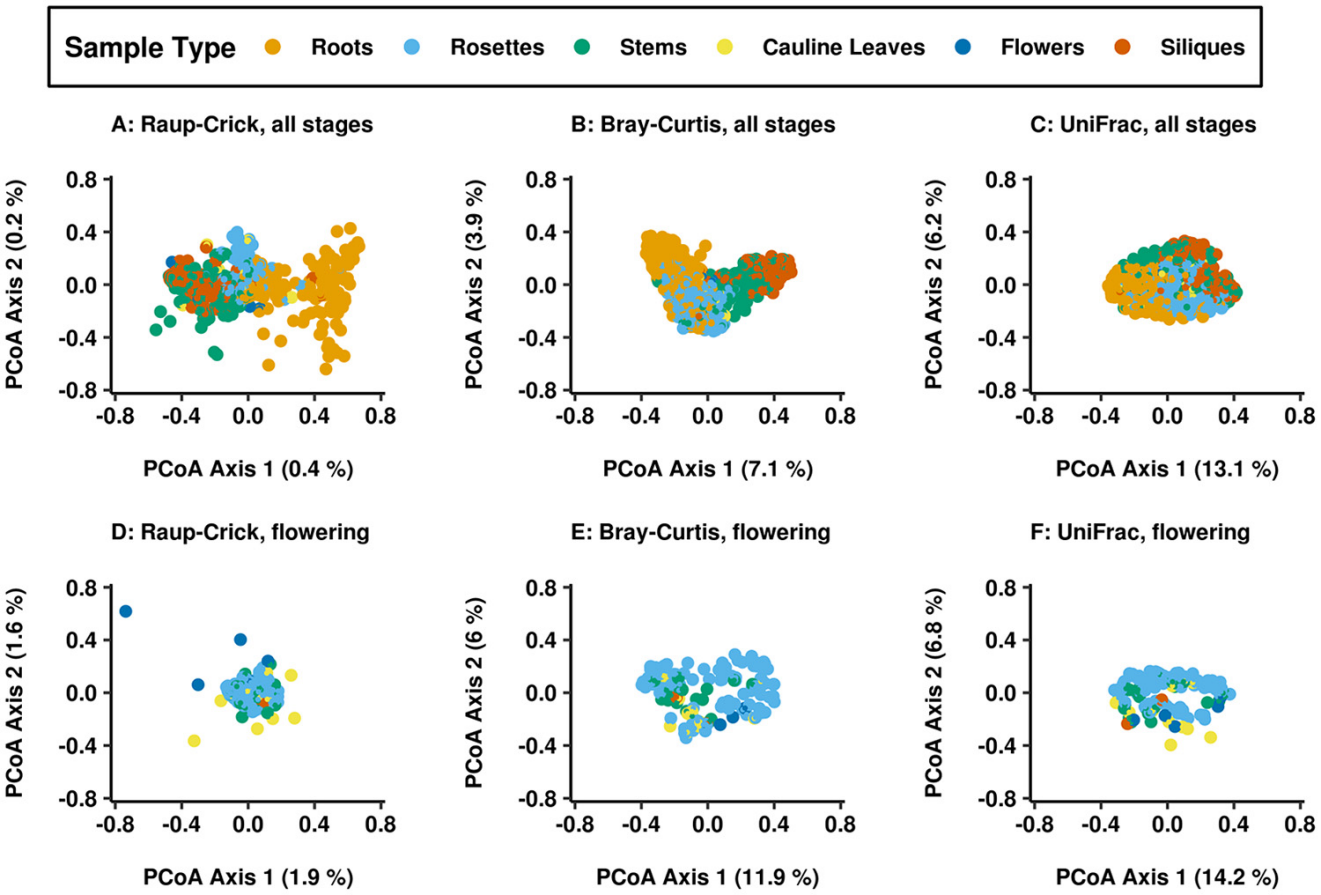
Roots and phyllosphere tissues housed distinct assemblages, while tissues within the phyllosphere segregated between but not within stages. Plant samples segregated by tissue type in principal-coordinate analysis (PCoA) based on their dissimilarities. The percentage of sample variance captured by the first two principal coordinates are listed on the *x* and *y* axes. (A and D) Raup-Crick dissimilarities are based on presence-absence differences between samples. (B and E) Bray-Curtis dissimilarities are based on quantitative differences in ASV counts between samples. (C and F) UniFrac distances incorporate phylogenetic relatedness of the ASVs present in samples based on the 16S gene tree. (A to C) For all dissimilarities, samples from roots (orange), rosette leaves (blue), and stems and siliques (green and red) clustered along the first coordinate. (D to F) In phyllosphere samples taken at flowering, rosette leaves (blue) overlapped with other phyllosphere tissues.

Assemblages varied more between root and shoot tissues than within the phyllosphere. In principal-coordinate analysis (PCoA) based on their dissimilarities (Fig. 2A to C), samples from the stem and siliques clustered separately from rosette leaf samples and from root samples. This finding was robust to differences in rarefaction depth, filtering, and normalization of the count data (44) (Fig. S1). However, segregation along the first two principal coordinates was not clear when phyllosphere samples from flowering plants were examined alone, suggesting that most of the association with tissue type was driven by differences between root and shoot (Fig. 2D to F). Supporting this interpretation, PERMANOVA on Raup-Crick dissimilarities of phyllosphere samples at flowering yielded a *P* value below the significance threshold (α = 0.001) (Table S4).

10.1128/mBio.02723-20.1 FIG S1 Plant tissue was the variable most strongly associated with sample composition, regardless of how composition variation was quantified or how the count tables for samples were transformed or thresholded. The relationships of host tissue type and stage to community composition were visualized by principal-coordinate analysis (PCoA) ordination of the samples based on dissimilarities or distances. Samples clustered primarily by the material sampled regardless of the dissimilarity or distance used. Observed tissue differences were robust to common methods for minimizing the effects of different count totals among samples. (A) Weighted UniFrac is influenced by the phylogenetic relationships of ASVs present in samples as well as quantitative differences in their abundances. This distance was calculated for a rarefied dataset. (B) Bray-Curtis dissimilarity is based on quantitative differences in ASV abundance across samples. This dissimilarity was calculated for relative abundances in all samples, without rarefaction. A variance-stabilizing transformation (C) or a log transformation (D) was performed on the count table before the Bray-Curtis dissimilarity was taken. Finally, the importance of host variables relative to site and year was robust to thresholding the ASV count table based on the prevalence or abundance. (E) ASVs were filtered for prevalence to include only those present in more than three samples. (F) ASVs were filtered for abundance to include only those with a count total exceeding 1,000 for all samples. Download FIG S1, TIF file, 0.6 MB. Copyright © 2021 Beilsmith et al. 2021 Beilsmith et al. This content is distributed under the terms of the Creative Commons Attribution 4.0 International license.

10.1128/mBio.02723-20.6 TABLE S4 PERMANOVA results for flowering phyllosphere samples. Download Table S4, PDF file, 0.03 MB. Copyright © 2021 Beilsmith et al. 2021 Beilsmith et al. This content is distributed under the terms of the Creative Commons Attribution 4.0 International license.

**FIG 3:**
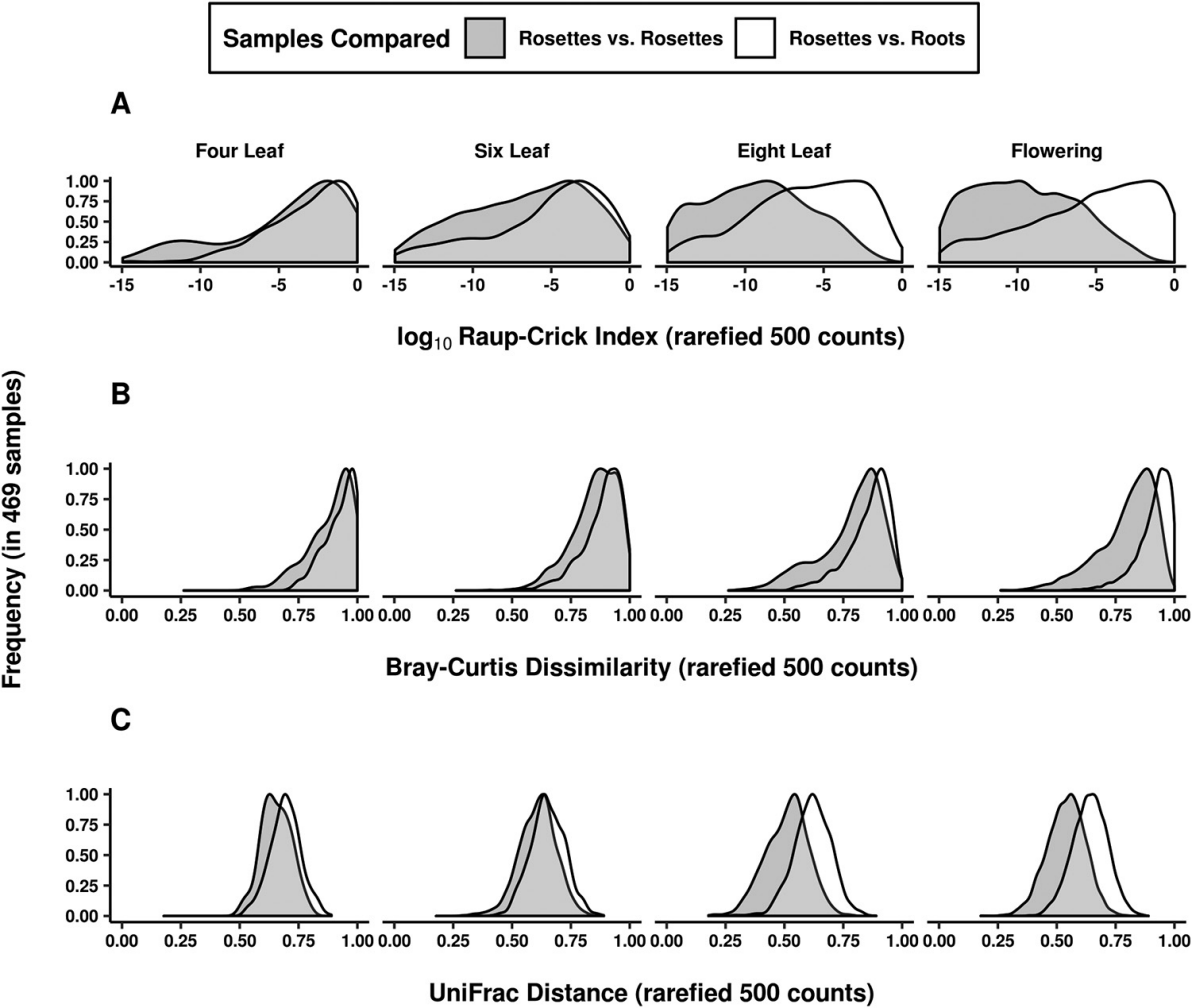
Phyllosphere assemblages became more distinguishable from those in roots as plants matured. The data set was pruned to samples with at least 100 counts and 20 ASVs to calculate sample dissimilarities. The distribution of dissimilarities between rosette leaf samples (shaded distribution) was compared to the distribution of dissimilarities between rosette leaf and root samples (unshaded distributions). This procedure was repeated for (A) Raup-Crick dissimilarities, (B) Bray-Curtis dissimilarities, and (C) UniFrac distances. As plants matured (left to right across each panel), phyllosphere samples were increasingly similar to each other and distinguishable from the root samples.

To disentangle the roles of tissue and age in defining habitats within the plant, we compared root and shoot tissues with respect to the ASVs present both before and after developmental transitions. Roots and rosettes were compared between late vegetative and flowering stages, while roots and stems were compared between flowering and senescence. Tissue assemblages grew more distinguishable later in development, with the proportion of variance explained by tissue relative to other host variables increasing at later stages (Table 3). The differentiation of tissue habitats over time was further examined by quantifying their β diversity at each stage. Pairwise dissimilarities of samples within and between tissue types were calculated and the distributions of these distances were compared for both rosette leaves and roots (Fig. 3). As development progressed, leaf assemblages simultaneously became more similar to each other and more distinct from those in the roots, perhaps due to unique selective pressures or a more restricted pool of potential colonists in the phyllosphere.

**TABLE 3:** PERMANOVA results for tissue comparisons at different stages

Stages	No. of samples	Tissue *R*^2^	Tissue Pr (>*F*)
Vegetative roots and rosettes	69	0.187	0.013
Flowering roots and rosettes	163	0.621	<0.001
Flowering roots and stem	94	0.391	<0.001
Senescent roots and stem	216	0.881	<0.001

### Assemblage associations with tissue and stage depended on subgenus variation in bacteria.

In the study of microbiome assembly, patterns can depend upon the taxonomic resolution with which bacteria are surveyed. ASVs represent the finest resolution of bacterial lineages possible with 16S data. If the effect of host tissue type was driven by differential filtering of closely related variants rather than larger bacterial clades, then it should weaken as taxonomic resolution decreases. To determine the dependence of the observed associations on taxonomic grouping, PERMANOVA was repeated with a Raup-Crick dissimilarity matrix produced with tables of ASV counts aggregated at the level of genus, family, order, class, and phylum (Table 4).

**TABLE 4:** PERMANOVA results for different taxonomic groupings

Taxonomic rank	% of ASVs unassigned	Tissue		Stage	
		*R*^2^	Pr (>*F*)	*R*^2^	Pr (>*F*)
ASV	0	0.803	<0.001	0.461	<0.001
Genus	43.94	0.769	<0.001	0.209	0.002
Family	17.71	0.682	<0.001	0.146	0.132
Order	8.17	0.202	0.003	0.155	0.004
Class	2.97	0.172	<0.001	0.081	0.065
Phylum	0.57	0.248	<0.001	0.153	0.001

Associations between assemblage composition and host tissue and age weakened with coarser taxonomic groupings of ASVs from genera to phylum. The weakening associations with coarser grouping suggested that the distribution of colonists within the host was not driven by widely shared bacterial traits but rather by variation within genera, whether acquired by horizontal transfer or evolved in vertically inherited traits. As a consequence, differences in the colonization patterns across tissues and stages are erased upon averaging the prevalence patterns of recently diverged lineages across taxonomic groups.

### Assemblage associations with tissue and stage were largely driven by the same ASVs, which were neither tissue specific nor transient.

By sampling different tissues at multiple time points, we were able to compare the bacterial lineages that distinguished assemblages across space and time. Spatial and temporal trends in the data were largely driven by the same colonists. To find ASVs significantly associated with specific tissues and developmental stages (α = 0.01), we compared the indicator value index of each ASV to a distribution generated by randomly permuting its presence-absence table (45). We found 460 ASVs that were significantly associated with specific tissue types; 70% of these were associated with roots, and the remainder were associated with different phyllosphere tissues. Of the 268 stage-discriminating ASVs, 76% were also among the 460 that distinguished tissue types.

Assemblage differences between tissues were not driven by specialists, and assemblage differences over time were not driven by transient community members. The ASVs associated with a specific tissue generally appeared in multiple plant tissues taken from a site in a given year rather than being restricted to a single habitat within the plant (93%). The ASVs associated with a specific stage generally recurred in a tissue throughout development rather than appearing at a single harvest stage (91%). Thus, assemblage differences did not result from the exclusive presence of ASVs in specific tissues or at specific stages in development, but rather from quantitative differences in prevalence over space and time.

### About a fifth of ASVs had consistent prevalence patterns across field sites and years, while the rest had inconsistent spatial and temporal distributions.

**FIG 4:**
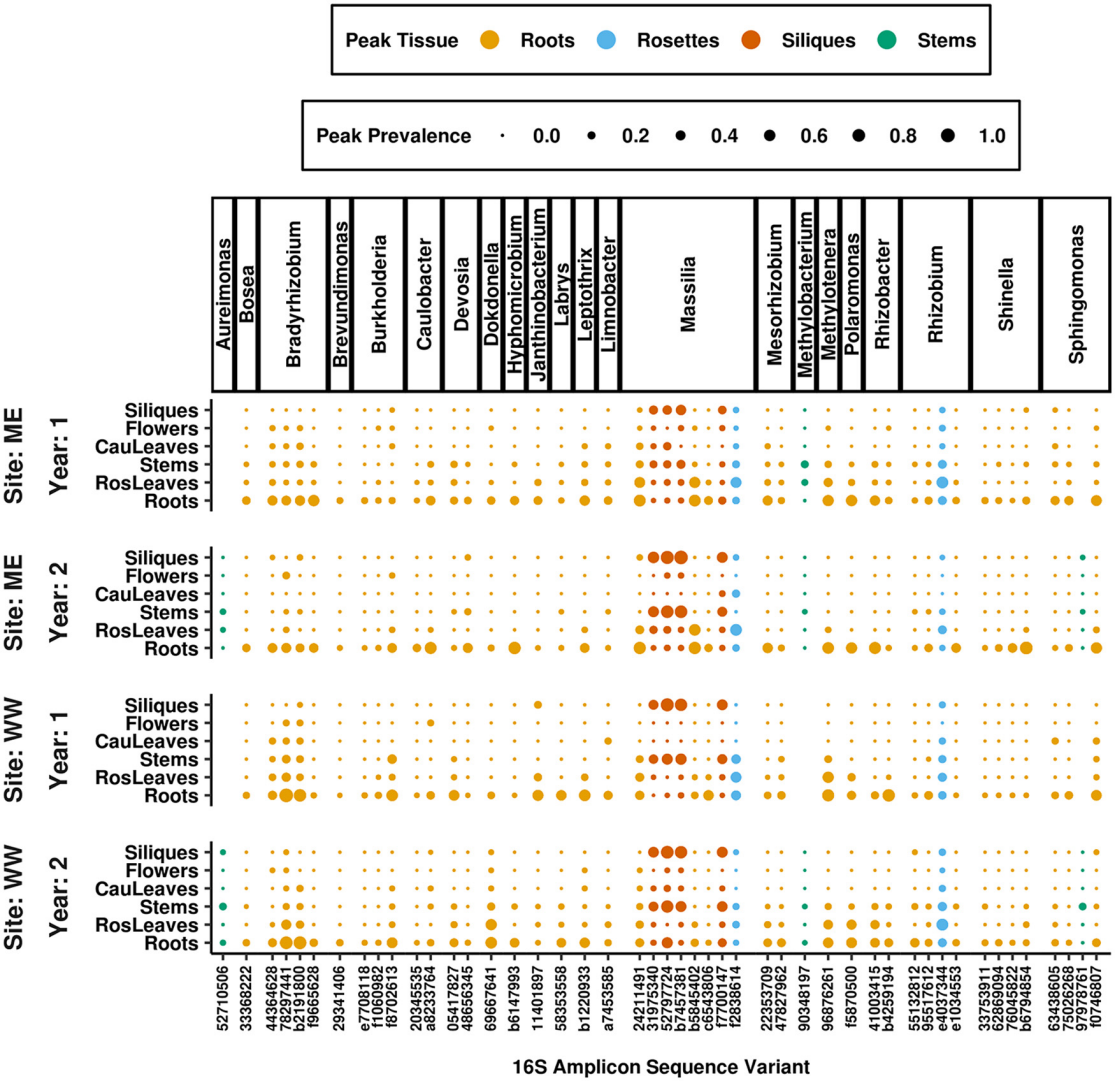
A fraction of ASVs behaved consistently across tissues at each site and in each year of the study. The ASVs distinguishing tissues, selected by indicator value indices, were filtered for those that reached maximum prevalence in the same tissue at each site and in each year when they were present. Proteobacteria ASVs with a total prevalence above 5% in all site and year combinations were selected for the plot. Panels on the *y* axis separate sites and years, and ASVs are grouped on the *x* axis by genus. Dot sizes represent the maximum prevalence for each ASV in each tissue. Despite the significant association detected between assemblage composition and tissue type, only 21% of tissue-discriminating ASVs consistently reached peak prevalence in the same tissue. Of the ASVs that behaved consistently, 79% always reached peak prevalence in roots.

**FIG 5:**
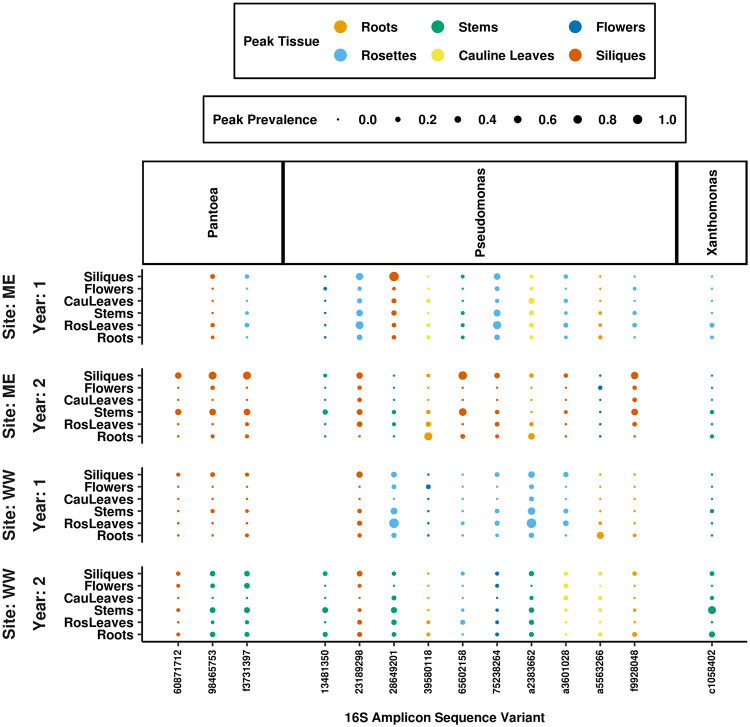
Most ASVs, including those in genera with pathogenic potential, do not have consistent spatial distributions across sites and years. The majority of ASVs distinguishing tissues did not reach maximum prevalence in the same tissue at each site and in each year when they were present. This pattern is exemplified by ASVs in the genera *Pseudomonas*, *Xanthomonas*, and *Pantoea*, which are notable for their pathogenic potential. Panels on the *y* axis separate sites and years, and ASVs are grouped on the *x* axis by genus. Dot sizes represent the maximum prevalence for each ASV in each tissue.

If tissue-specific host traits created environments favorable to particular colonists, then the spatial distributions of those colonists within the plant should be repeated across the sites and years in which they were observed. To assess whether ASV spatial distributions were repeated, the tissues in which ASVs reached maximum prevalence, when present, were compared between sites and years. Based on these comparisons, 21% (98/460) of the ASVs distinguishing tissues displayed consistent spatial trends, always peaking in prevalence in the same tissues (shown for *Proteobacteria* in Fig. 4). For this fraction of ASVs, colonization patterns might be linked to tissue-specific host traits that differentially filter bacterial colonists. Of these consistently distributed ASVs, 79% were always most prevalent within roots, 11% in rosettes, 5% in stems, and 5% in siliques. Notably, the genus *Massilia* includes two distinct sets of ASVs that consistently peaked in different tissues; one set consistently peaked in roots, while the second consistently peaked in siliques, emphasizing that subgenus variation between bacterial lineages influenced their distributions within plants. For the remaining ASVs, including those in notable pathogen genera (Fig. 5), spatial prevalence patterns were inconsistent between sites and years.

**FIG 6:**
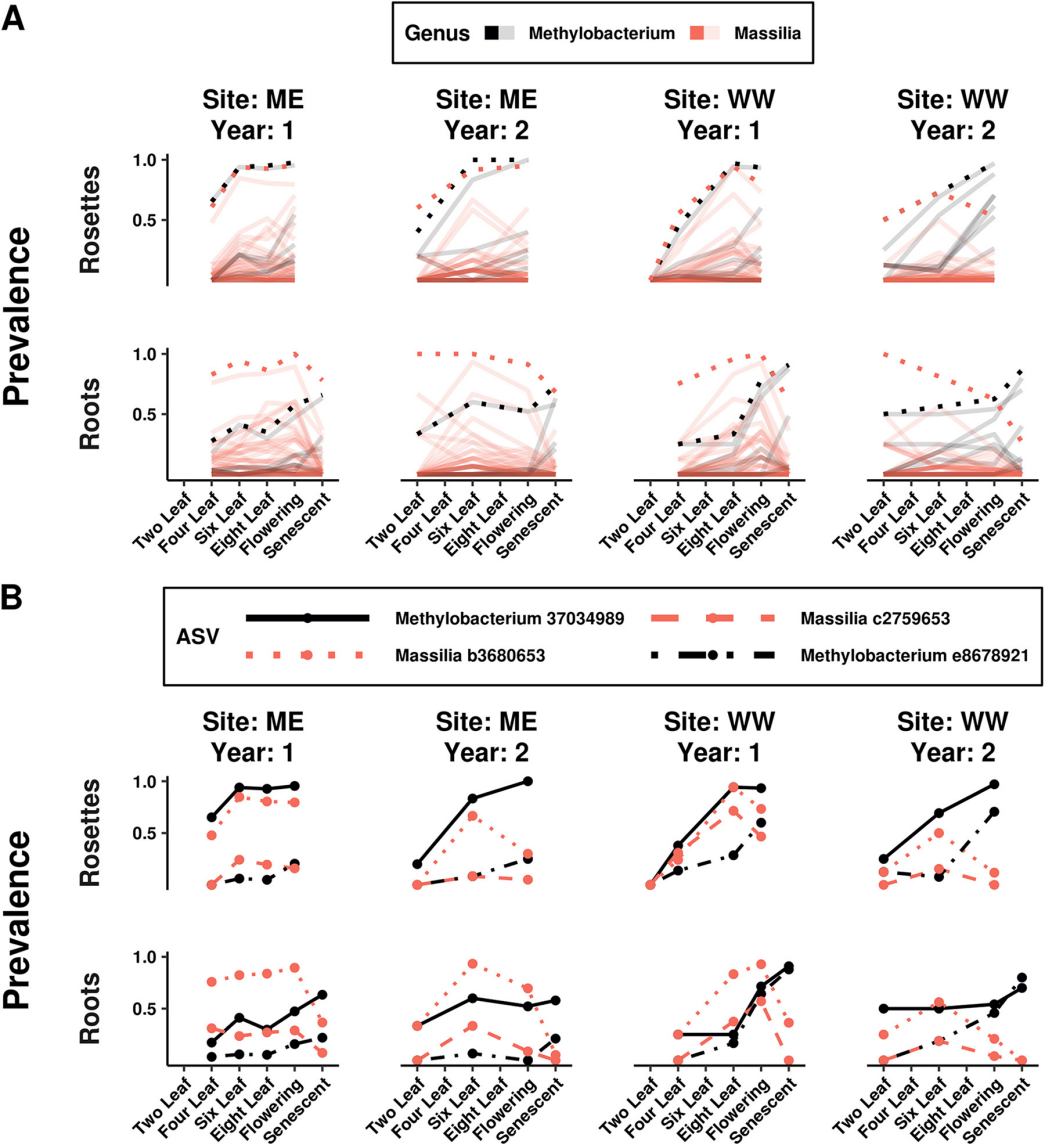
ASVs in the same genus have distinct temporal trends; a small number consistently reach high prevalence across sites and years despite variation in temporal trends. Plots feature *Proteobacteria* genera (*Massilia* in red and *Methylobacterium* in black) containing ASVs that distinguished developmental stages, based on indicator value indices, and reached over 70% prevalence in multiple sites and years. Horizontal panels separate the trends for roots and rosettes, and vertical panels separate the trends for each site and year. (A) Bold trend lines show the temporal trends for counts grouped by genus, while transparent lines show the trends for individual ASVs in each genus. Genus-level trend lines subsume distinct ASV patterns. (B) Temporal colonization patterns were highly inconsistent despite the significant association between composition and stage, with only 23% of stage-discriminating ASVs consistently peaking at the same stage. Despite this variation, specific *Massilia* and *Methylobacterium* ASVs were among the most prevalent at both sites and in both years of study.

Temporal distributions, like spatial ones, were highly variable. Of the ASVs driving associations with stage, only 23% (62/268) reached peak prevalence at a consistent plant developmental stage across sites and years. Temporal distributions were also dependent on fine taxonomic variation, because ASVs within each genus displayed a variety of dynamics (Fig. 6A). Among the *Proteobacteria* ASVs driving associations with age, the ones demonstrating the biggest changes in prevalence were found in both roots and rosettes across sites and years (Fig. 6B). Temporally dynamic *Massilia* ASVs peaked during vegetative growth or flowering and then declined. Temporally dynamic *Methylobacterium* ASVs consistently increased during plant growth and peaked at senescence.

### Assemblages became more phylogenetically diverse and even over time.

**FIG 7:**
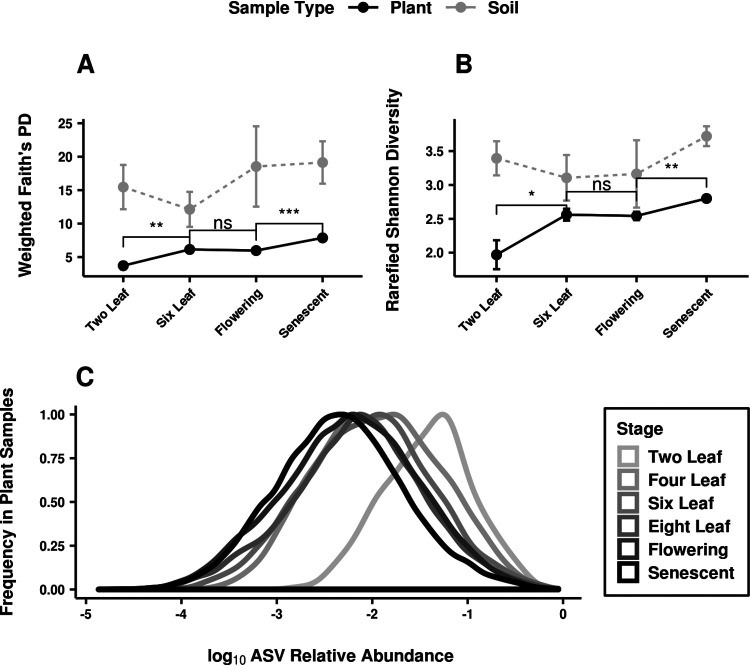
Assemblages within plants became more phylogenetically diverse and even over time. (A and B) Plots show means and standard errors for diversity measures of the plant (black) and soil (gray) samples at each developmental stage in the second year of study. Significance is shown for pairwise Wilcoxon tests between stages as follows: ns, not significant; *, *P* < 0.05; **, *P* < 0.01; ***, *P* < 0.001; ****, *P* < 0.0001. (A) Phylogenetic distance was measured as the branch length on the 16S phylogenetic tree between ASVs in the sample, weighted by ASV abundances. It increased on average with developmental stage. (C) Shannon-Wiener (H′) indices from rarefied samples depended upon both the richness and evenness of samples and increased on average with developmental stage. (C) ASV relative abundances decrease during development as assemblages become more even. For plant samples with at least 100 counts, the relative abundance of each ASV present was calculated. The frequency distributions of these relative abundances are plotted for each developmental stage (shade), with relative abundance scaled by log_10_.

Temporal changes in assemblage α diversity can help explain the increased differentiation between assemblages inhabiting different tissues. The phylogenetic diversity of colonists was higher later in plant development (Fig. 7A). Phylogenetic diversity was quantified by taking the tree of 16S variants present in each assemblage, weighting the branch lengths by variant abundance, and summing the branch lengths (46). This diversity trend was observed in each type of plant tissue sampled (Fig. S2) but not in samples of the surrounding soil, indicating that it was related to the colonization of plant tissue and not purely driven by the abiotic environment during sample collection. The increasing phylogenetic diversity suggested that bacteria from across the tree had dispersed more widely among plant assemblages later in development. With more opportunities to encounter plants over time, subtle differences in the ASV colonization success between tissues were more likely to be exposed.

10.1128/mBio.02723-20.2 FIG S2 Diversity and evenness increased during development in all plant tissues. All plots show means and standard errors for diversity measures of the plant samples at each developmental stage in the second year of study. Flower and cauline leaves were sampled at only one developmental stage and thus do not have trend lines. (A) Phylogenetic distance was measured as the branch length on the 16S phylogenetic tree between ASVs in the sample, weighted by ASV abundances. It increased on average with developmental stage. (B) Shannon-Wiener (H′) indices from rarefied samples depended upon both the richness and evenness of samples and increased on average with developmental stage. Download FIG S2, TIF file, 0.1 MB. Copyright © 2021 Beilsmith et al. 2021 Beilsmith et al. This content is distributed under the terms of the Creative Commons Attribution 4.0 International license.

Temporal trends in assemblage evenness suggested that tissue type was related to the size of the colonization bottleneck for specific ASVs rather than to their ability to dominate other colonists once established in the endosphere. Variants that reached high prevalence in a tissue did not overtake assemblages in terms of relative abundance. The average Shannon-Wiener index (47) of assemblages increased (Fig. 7B) and the distribution of ASV relative abundances decreased (Fig. 7C) during the life of the plant. Together, these trends showed that instead of domination by a small number of successful variants, mature tissues on average housed a more evenly represented set of ASVs.

## DISCUSSION

We surveyed bacterial assemblages inside roots and phyllosphere tissues throughout the life cycle of the model annual plant *A. thaliana*. Consistent with previous results, we found that assemblage composition differed between tissues (29) and during the course of development (20, 30). Because we examined multiple tissues and developmental stages in the same study, we were able to find three connections between these spatial and temporal trends in natural colonization that have not previously been identified.

First, the associations of host tissue type and developmental stage with assemblage composition were largely driven by the same colonists. These bacterial variants did not constitute omnipresent cores of tissue-specific inhabitants: those most strongly associated with tissue type typically did not reach even 50 percent prevalence within samples from a tissue type at a given time, underscoring the variability of community composition. Nor were these variants exclusive to specific habitat patch within the plant: most were observed at multiple sampling times and in multiple tissues. Instead, differential filtering over space and time quantitatively affected the prevalence of common endophytes, in agreement with a recent report of consistent organ occupancy in the plant microbiome (24).

Second, endophytic assemblages filtered by root and shoot tissues became more distinguishable later in development. Specifically, leaf assemblages grew more differentiated from root assemblages on average. ASVs with consistently higher prevalence in specific tissues suggested the existence of subtle differences between variants in the probability of colonizing different tissue niches. Increasing phylogenetic diversity indicated that bacterial lineages became more widely dispersed during plant development. As variants had more opportunities to colonize plant tissue, differences in the probability of colonizing particular tissues became more important in determining assemblage composition than the stochasticity of early colonization. Supporting this idea of the host plant gaining influence during assembly, a recent study of rice (Oryza sativa) roots found that microbiome composition was dynamic during vegetative growth and stabilized later in plant life (48).

A third major pattern in our data is the variability of spatiotemporal distributions among lineages within the same bacterial family or even genus. Most ASVs associated with tissue type (70%) were significantly enriched or depleted in the roots. Indeed, many of these ASVs belonged to genera that were consistently detected as root endophytes in a study of 17 European sites over 3 years (49). These included members of *Massilia*, *Burkholderia*, and *Bradyrhizobium*. However, *Massilia* also contained ASVs that reached peak prevalence in rosette leaves or siliques. An important factor in detecting associations between host and colonists is the resolution with which bacterial lineages are grouped in count tables. When microbes derived from natural sources, including plant leaves, are passaged outside the host in minimal medium, they produce communities that are similar at the family level despite being highly variable at the level of sequence variants (50). In our study, grouping variants by family weakened associations with tissue type and erased the observed associations with harvest stage. Unlike the carbon metabolism traits that determined community structure *ex situ* (50), the functions selected by different tissue habitats may therefore not be shared broadly by lineages in a family.

In contrast to the roots, the phyllosphere presents microbes with a variety of challenges related to desiccation, toxins from other colonists, and motility (51). Adaptations known to mitigate these challenges, such as chemosensory and antimicrobial resistance genes, have occurred recently and vary at the subgenus level in the lineages of known phyllosphere colonists (52, 53). Traits like these could lead to greater success in colonizing the leaf niche, creating phyllosphere assemblages that are more similar to each other and distant from root assemblages. As a result, closely related strains of known pathogenic, plant-beneficial, or biocontrol taxa might not be established in the same way throughout their plant hosts. Functional profiling of the bacterial assemblages may therefore be more valuable than taxonomic profiling in understanding their spatial and temporal trends within plants.

Our experimental design, including replicate sites and study years, allowed us to characterize consistency in distributions over space and time for the variants associated with host plant features. Biotic factors that can differ between roots and the phyllosphere, like salicylic acid, have been manipulated and shown to influence community assembly in both lab and field conditions (54, 55). If such deterministic factors drove the observed variation in composition, then we might expect to see bacterial endophytes with the same across-tissue spatial distributions in repeated surveys. However, only about 20% of variants distinguishing tissues were consistently more prevalent in a specific tissue. The inconsistent behavior of most variants is perhaps not surprising given that samples typically did not share more than 15% of their colonists. This variability in the detection of endosphere colonists suggests that in nature, as in more controlled environments (17), stochastic factors drive much of community assembly. Even if individual lineages interact consistently with host plants, chance entry of functionally redundant strains from a large pool of soil colonists may give rise to mosaic assemblages.

Despite the growing strength of tissue effects during development, assemblages did not collapse to a few successful inhabitants of each tissue. In contrast, assemblage evenness increased in all plant tissues, agreeing with previous reports that community diversity increases as host plant tissues age (30, 31). While host tissues appear to create different colonization bottlenecks for bacterial lineages, they did not appear to favor the dominance of these ASVs over others within assemblages.

These findings have consequences for the study of another key determinant of plant microbial communities: host genotype. Host genotype effects on colonization efficiency and microbiome composition are found both among angiosperm species that diverged hundreds of millions of years ago and among crop accessions that diverged through domestication within 10,000 years (19, 26, 56–59). Compositional differences in the field can even be related to polymorphisms within a plant species (4, 60). Since the variants associated with host variables are typically present throughout the plant and recurrent during development, filtering complex natural assemblages with these criteria can increase signal in the search for host polymorphisms linked to colonization success. Since tissue-associated assemblages become more distinct later in development, the host polymorphisms linked to variant prevalence or abundance might depend on which part of the plant is sampled, and when.

The effects of plant tissue type and developmental stage on assemblage composition were large compared to that of geographic site. Our results add to a growing body of evidence that the tissue sampled from a plant can explain more variation in microbial communities than geography. Studies of *A. thaliana* and Boechera stricta have found that geographic sites and soil inocula play a substantial role in filtering plant microbial communities (20, 23). However, studies of cultivated *Agave* have found that tissue explains more variation in community composition than species and site (27). Tissue type also explains more variation in epiphytic community composition than sample site in the species Scaevola taccada (28). Together, these results suggest that plants are best considered as collections of distinct habitat patches for bacterial colonization.

## MATERIALS AND METHODS

### Planting.

Field experiments were replicated over 2 years (2012 to 2014) at two locations: Michigan State Southwest Michigan Research and Extension Center (ME) and University of Chicago Warren Woods Ecological Field Station (WW). Prior to planting in October, fields were tilled and grids were created with bottomless plastic pots (6 to 12 cm across) placed 2 to 5 cm into the ground and 10 to 30 cm apart. Within each grid, seeds for seven midwestern *A. thaliana* ecotypes were sown randomly and a fraction of pots were left empty for soil sampling. Seeds were surface sterilized with ethanol, and seedlings were thinned after germination with sterilized tweezers.

### Sample collection.

Plant sampling order was randomized and all tools were flame sterilized with ethanol between samples. Root and above-ground tissues were separated into sterile plastic tubes. For soil samples, sterile tubes were pushed 2 to 5 cm into the ground. Tubes were stored at −80°C until processing. To remove loosely associated microbes, each plant sample was washed twice by vortexing with surfactant buffer (22). Plant samples were then transferred to Matrix tubes (Thermo Scientific, Waltham, MA, USA). Aboveground tissue was first separated into compartments with a scalpel and tweezers. For large tissues, only enough material was added to allow bead homogenization. For soil, samples were put through a 2-mm sieve and ∼100 mg was transferred to a Matrix tube. The tubes were randomized in 96-well racks with respect to sampling site, year, and time point. To dry the material, tubes were frozen to −80°C and lyophilized overnight. To powder the tissue, sterile silica beads were sealed into each tube with a SepraSeal cap (Thermo Scientific) and tubes were shaken on a 2010 Genogrinder homogenizer (SPEX, Metuchen, NJ, USA) (1,750 rpm, 2 min). Dry mass was recorded, and up to 36 mg of material was retained per tube. All tubes were then randomized in Nunc 96-well DeepWell plates (Thermo Scientific) for DNA extraction.

### DNA extraction.

Ground material was resuspended in TES (10 mM Tris-Cl, 1 mM EDTA, 100 mM NaCl) to a concentration of 0.04 mg/μl. Material was homogenized with a Genogrinder (1,750 rpm), and homogenates (240 μl) were incubated (30 min) in new plates with lysozyme solution (Epicentre, Madison, WI, USA) at a final concentration of 50 U/μl. Proteinase K (EMD Millipore, Billerica, MA, USA) and SDS were added to final concentrations of 0.5 mg/ml and 1%, respectively. Plates were incubated at 55°C for 4 h. An equal volume of 24:1 chloroform-isoamyl alcohol was mixed by pipette in each well. Plates were centrifuged at 6,600 × *g* with the Beckman Coulter Avanti J-25 centrifuge (Beckman Instruments, Munich, Germany) for 15 min at 4°C. The top aqueous layer (350 μl) was removed and added to new plates with 500 μl 100% isopropanol. Plates were inverted to mix and incubated 1 h at −20°C. After centrifugation for 15 min at 4°C, isopropanol was removed and DNA pellets were washed with 500 μl 70% ethanol. Pellets were dried in a chemical hood and resuspended in TE (100 μl; 10 mM Tris-Cl, 1 mM EDTA) by shaking. After incubation on ice for 5 min, plates were centrifuged for 12 min at 4°C, and supernatants diluted 10× in TE were added to new 0.5-ml plates for PCR amplification.

### 16S rRNA gene amplification.

The V5, V6, and V7 regions of the 16S rRNA gene were amplified from each sample using the 799F and 1193R primers with Illumina MiSeq adapters and custom pads, linkers, and barcode sequences (61). The PCR volume was 25 μl: 1 μl of 10× diluted DNA template, a 0.2 μM concentration of each primer, 1× 5PRIME HotMasterMix (5PRIME, Gaithersburg, MD, USA), and 0.8× SBT-PAR additive (5× stock: 750 mM sucrose, 2 mg/mL BSA, 1% Tween-20, 8.5 mM Tris-Cl pH 7.5) (62). PCR amplification consisted of initial denaturation at 94°C for 2 min, followed by 35 cycles of denaturation at 94°C for 30 s, annealing at 54.3°C for 40 s, and elongation at 68°C for 40 s, followed by a final elongation at 68°C for 7 min. Each PCR was completed in triplicate, and products were pooled and purified with an equal volume of Axygen AxyPrep Mag PCR Clean-Up bead solution (Corning, Tewksbury, MA, USA). Amplicon concentrations were quantified by fluorimetry (QUANT-iT PicoGreen double-stranded DNA [dsDNA] assay kit; Life Technologies, Carlsbad, CA, USA) and 30 ng or a maximum of 30 μl per sample was pooled for six plates per sequencing run. Primer dimers and mitochondrial amplicons were removed by concentrating each amplicon pool 20× (Savant SPD121P SpeedVac concentrator; Thermo Scientific) and purifying the 300- to 700-bp product with BluePippin (Sage Science, Beverly, MA, USA).

### Sequence data.

Amplicon pools were sequenced using the Illumina MiSeq platform and MiSeq V2 reagent kits (Illumina, San Diego, CA, USA) to produce paired-end 250-bp reads (MiSeq Control software v2.5.0.5). MiSeq Reporter v2.5.1.3 demultiplexed samples and removed reads without an index or matching PhiX. Within QIIME2, cutadapt removed primers from the paired reads and DADA2 identified ASVs. Primers 799F and 1193R were used to extract reads *in silico* from the QIIME-SILVA 16S database. These reads were used to build a classifier using QIIME2’s naive-bayes method, and the sklearn algorithm was used to generate taxonomy assignments for the sequence variants. These assignments were used to filter any remaining mitochondrial and chloroplast sequences. Sequence variants with a frequency lower than 2 counts, samples with fewer than 10 reads, and samples with notes on irregularities during collection were also removed. To generate a phylogeny for the sequence variants, QIIME2 was used to align the sequences with MAFFT and to infer and root a phylogenetic tree. The tree was imported along with the DADA2-generated ASV count table, the taxonomy, and the metadata into a phyloseq (63) class in R (version 3.4.4) (64) for analysis. Count table transformations, pruning, and rarefaction were performed with phyloseq, and distance matrix calculation, ordination, and PERMANOVA tests were performed with the vegan package (65). Phylogenetic analysis was performed with ape and picante (66, 67). Figures and supplemental figures were produced with ggplot2 and ggpubr (68, 69).

### Statistics.

Three dissimilarity metrics were used to capture different aspects of microbiome variation. Presence-absence variation was represented by the Raup-Crick dissimilarity index, a probability of samples differing in composition based on ASV frequencies in the data set. Alternatively, the Bray-Curtis dissimilarity quantified the abundance differences between ASV counts in each sample. The UniFrac distance incorporated presence-absence variation as well as phylogenetic relatedness between the ASVs present in samples based on the 16S gene tree.

ASVs associated with specific tissues or developmental stages were identified using the signassoc function of the indicspecies package (45, 70). This function calculated an indicator value index (IndVal) based on the product of two probabilities: (i) the probability that a sample belonged to a habitat given ASV presence and (ii) the probability that an ASV was present if a sample was taken from a habitat. For the habitats defined by each variable (six tissues, six developmental stages, two sites, and two years), indices were calculated independently for each ASV. The null hypothesis that no relationship existed between ASVs and conditions was tested by comparing the empirical index with a distribution generated by randomly permuting the ASV presence-absence count table. A two-tail *P* value was used to select ASVs that are significantly more or less frequently observed in sampled belonging to a given condition (α = 0.01).

### Data availability.

Raw sequencing data are available in the NCBI's Sequence Read Archive, BioProject ID PRJNA607544. The ASV count table, 16S phylogenetic tree and taxonomy, and sample metadata are available with the R commands used for analysis at https://github.com/krbeilsmith/KBMP2020_Microbes.
