# Supplementary material for: Natural Bacterial Assemblages in Arabidopsis thaliana Tissues Become More Distinguishable and Diverse during Host Development
Source: mBio. 2021 Jan 19;12(1):e02723-20. doi: 10.1128/mBio.02723-20 (PMC7845642; doi:10.1128/mBio.02723-20)
Supplement: TABLE S1 [file mBio.02723-20-st001.pdf]

TABLE S1 Arabidopsis thaliana ecotypes planted in the study (from the HPG-1 haplogroup)

Accession	Ecotype ID	Collection Location	Coordinates
BRR4	470	Watseka, IL, USA	(40.8313, - 87.735)
LI-WP-041	546	Nissequogue, NY, USA	(40.9076, - 73.2089)
L-R-10	1797	Union Pier, MI, USA	(41.847, - 86.67)
MNF-Che-47	1942	Manistee National Forest, MI, USA	(43.5251, - 86.1843)
Pent-7	2191	Pentwater, MI, USA	(43.7623, - 86.3929)
PT1.85	8057	Hanna, IN, USA	(41.3432, - 86.7368)
SLSP-69	2285	Silver Lake, MI, USA	(43.665, - 86.496)
