# Supplementary material for: Natural Bacterial Assemblages in Arabidopsis thaliana Tissues Become More Distinguishable and Diverse during Host Development
Source: mBio. 2021 Jan 19;12(1):e02723-20. doi: 10.1128/mBio.02723-20 (PMC7845642; doi:10.1128/mBio.02723-20)
Supplement: TABLE S2 [file mBio.02723-20-st002.pdf]

TABLE S2 Sample collections for the study

Year	Date	Site	Stage	Sample type(s)
1	10/12/12	ME	No Plant	Soil
1	10/15/12	WW	No Plant	Soil
1	10/26/12	ME	Two Leaf	Roots, Rosette Leaves
1	10/29/12	WW	Two Leaf	Roots, Rosette Leaves
1	11/29/12	ME,WW	Four Leaf	Roots, Rosette Leaves
1	02/13/13	ME	Six Leaf	Roots, Rosette Leaves
1	03/29/13	ME, WW	Eight Leaf	Roots, Rosette Leaves
1	05/01/13	ME, WW	Flowering	Roots, Rosette Leaves, Stems, Cauline Leaves, Flowers, Siliques
1	06/14/13	ME, WW	Senescent	Roots, Stems, Siliques
2	10/28/13	ME, WW	No Plant	Soil
2	12/04/13	ME, WW	Two Leaf	Soil, Roots, Rosette Leaves
2	04/16/14	ME, WW	Six Leaf	Soil, Roots, Rosette Leaves
2	05/15/14	ME, WW	Flowering	Soil, Roots, Rosette Leaves, Stems, Cauline Leaves, Flowers, Siliques
2	07/03/14	ME, WW	Senescent	Soil, Roots, Stems, Siliques
