# Supplementary material for: Natural Bacterial Assemblages in Arabidopsis thaliana Tissues Become More Distinguishable and Diverse during Host Development
Source: mBio. 2021 Jan 19;12(1):e02723-20. doi: 10.1128/mBio.02723-20 (PMC7845642; doi:10.1128/mBio.02723-20)
Supplement: TEXT S1 [file mBio.02723-20-s0001.pdf]

SUPPLEMENTARY MATERIALS AND METHODS
for "Natural bacterial assemblages in *Arabidopsis thaliana* tissues become more distinguishable and diverse during host development" by Beilsmith, Perisin, and Bergelson 2020

Field experiments were replicated over the course of two years (2012-13, 2013-14) and at two locations: Michigan State Southwest Michigan Research and Extension Center (ME) in Benton Harbor, MI, USA (42.0853, -86.3588) and University of Chicago Warren Woods Ecological Field Station (WW) in Three Oaks, MI, USA (41.8362, -86.63). Sites were tilled prior to each year's planting. In the first year, the ME site was planted on October 12, 2012 and WW site was planted on October 15, 2012. In the second year, both sites were planted on October 28, 2013 (1).

Planting
In the first year, the bottoms of 11.43 cm diameter plastic plots were removed before being placed 2-5 cm into the ground. Pots were placed 30.48 cm apart in a grid (ME: 100 rows by 8 columns with rows going north to south; WW: 50 rows by 15 columns with rows going north to south). In the second year, black landscaping cloth was placed at each site after tilling to reduce native weed and grass growth (ME: 19 days prior to planting; WW: 7 days prior to planting). On the day of planting, pot size holes were cut in the cloth, the bottoms of 6.35 cm square pots were removed, and pots were placed 2-5 cm into the ground. Pots were placed in blocks (4 blocks/site, 1.22 m between blocks going north to south) with each block containing a 16 row by 10 column grid with 10 cm between pots (rows going north to south).

In both years, surface sterilized seeds were spread in each pot and plant ecotypes, along with empty pots for soil samples, were completely randomized within each grid. Seven *A. thaliana* ecotypes were planted. All ecotypes were collected from the Midwestern United States (Supplementary Table S1) and belong to the near isogenic haplogroup-1 (HPG1) (2,3). These midwest accessions germinate in the fall, overwinter as small rosettes, flower in the spring, and senesce in the early summer. Seeds were surface sterilized in 1.5mL Eppendorf tubes by addition of 70% ethanol, incubation at room temperature for 1 minute, removal of ethanol, addition of 100% ethanol, incubation at room temperature for 1 minute, and removal of ethanol followed by drying in a sterilized biological cabinet. After germination, seedlings were thinned to 5-10 plants per pot and any native weed and grass was removed. Tweezers were ethanol sterilized between pots while thinning.

Sample collection
At each sampling time point, the time and surface soil (2-5 cm deep) temperature were recorded for each sample. The sampling order was randomized over the entire grid for Year 1 (8 replicates per ecotype per site per time point) and over each block for Year 2 (2 replicates per ecotype/empty pot per block per site per time point). For empty pot soil samples, a sterile 15 mL conical tube was pushed into the ground (2-5 cm), soil was collected, and the tube was capped and placed on dry ice. In a plastic tray, tweezers and a razor were used to remove root tissue from aboveground tissue. Each type of tissue was placed in a separate 15 mL conical tube. In between samples, the spatula, tweezers, and razor were flame sterilized with 100% ethanol, and the tray was sterilized with 100% ethanol. Tubes were placed on dry ice until transport back the University of Chicago. Tubes were stored at -80 C until processing.

Sample processing
All samples from a plant were kept together and plants were randomized with respect to sampling site, year, and timepoint across the 96-well plates. Prior to processing, all samples were kept at -80 C except during randomization. Ten plants were thawed and processed at a time. To remove loosely associated microbes, each plant sample was washed twice. For each root or above-ground sample, the tissue was

removed and added to a 50 mL conical tube with 25 mL of surfactant buffer (4) (6.33 g $NaH_2PO_4 \cdot H_2O$, 16.5 g $Na_2HPO_4 \cdot 7H_2O$, per 1 L, autoclaved then 200 µL Silwet L-77 added). Tubes were vortexed for 10 s then material was transferred to a new 50 mL conical tube with fresh 25 mL surfactant buffer. Tubes were vortexed again for 10 s.

Above-ground tissue was removed from buffer and placed in a glass tray to further separate plant compartments. Tweezers and a scalpel were used to separate tissues and to cut tissue into pieces small enough to fit into 1.4 mL Matrix tubes (Thermo Scientific, Waltham, MA, USA). Each root and above-ground tissue sample was placed into Matrix tubes in 96 well racks and sealed with SepraSeal caps (Thermo Scientific). For large tissues, only enough material was added to allow for bead homogenization. For each soil sample, soil was sieved through a 2 mm sieve onto a glass tray. A spatula was used to add ~100 mg of soil to the appropriate Matrix tube. Tweezers, spatula, scalpel, and glass tray were flame sterilized and the sieve was ethanol sterilized between samples.

Matrix tubes were placed at -80 C overnight. The next morning, caps were removed and plates were covered in parafilm. Over each tube, a small hole was punched and the Matrix plates were lyophilized overnight (LABCONCO FreeZone 4.5, Kansas City, MO, USA). Parafilm was then removed and tubes were sealed with new caps. Two 2.3 mm silica beads (BioSpec, Bartlesville, OK, USA) were added to each tube, and samples were ground to powder with a 2010 Genogrinder (SPEX, Metuchen, NJ, USA) (1750 RPM, 2 min). Dry mass was recorded and up to 36 mg of material was retained per tube. All tubes were then randomized over thirty 96 well DNA extraction plates, including empty well controls. Plates were kept at room temperature until DNA extraction.

DNA extraction

All following pipetting and shaking steps were completed using custom liquid handling robotics scripts on the Freedom Evo 200 (Tecan, Morrisville, NC, USA), unless indicated differently. All centrifugation steps were completed using the Beckman Coulter Avanti J-25 centrifuge (Beckman Instruments, Munich, Germany). Two 96 well plates were processed each day. To start, ground material was centrifuged at 6600xg for 2 min. Material was resuspended in TES (10 mM Tris-Cl, 1 mM EDTA, 100 mM NaCl) to a concentration of 0.04 mg/µL. A minimum of 250 µL TES was added per tube. Tubes were sealed with new caps and material was homogenized with the 2010 Genogrinder (SPEX) (two rounds of 2 min 30 s, 1750 RPM). Homogenates (240 µL) were manually transferred to new Nunc deepwell 96 well plates (Thermo Scientific). Ready-lyse lysozyme solution (Epicentre, Madison, WI, USA) was manually added to each well to a final concentration of 50 U/µL. Plates were vortexed for 10 s and incubated at room temperature for 30 min. After a flash centrifugation, proteinase-K (EMD Millipore, Billerica, MA, USA) and SDS were added to each well to final concentrations of 0.5 mg/mL (20 mg/mL stock in 50 mM Tris-Cl pH 8, 3 mM $CaCl_2$, 50% glycerol, filter sterilized through 0.2 µm pore filter) and 1%, respectively. Plates were vortexed for 10 s and incubated in a 55 C water bath for 4 h. After a flash centrifugation, an equal volume of 24:1 chloroform:isoamyl alcohol was manually added to each well and pipetted to mix. Plates were centrifuged at 6600xg for 15 min at 4 C. The top aqueous layer (350 µL) was removed and added to new deepwell plates with 500 µL 100% isopropanol per well. Plates were inverted 50 times to mix and incubated at -20 C for 1 h. After centrifugation at 6600xg for 15 min at 4 C, isopropanol was removed, and DNA pellets were washed with 500 µL 70% ethanol. Pellets were air dried in a chemical hood with a 96 well blower and resuspended in TE (100 µL, 10 mM Tris-Cl, 1 mM EDTA) by shaking for 4 min. After incubation on ice for 5 min, plates were centrifuged at 6600xg for 12 min at 4 C. DNA supernatants were removed from impurities in the pellets and added to new Nunc 0.5 mL 96 well both for storage (no dilution, kept at -20 C) and PCR amplification (10X dilution in TE, kept at 4 C).

PRIMERS FOR 16S RIBOSOMAL RNA GENE AMPLIFICATION AND SEQUENCING

799F (forward primer) PCR primer sequence:
Field number (space-delimited), description:
1. 5’ Illumina adapter
2. i5 index (Listed in Table B.1)
3. Forward primer pad
3. Forward primer linker
4. Forward primer

AATGATACGGCGACCACCGAGATCTACAC NNNNNNNN TACCCCCCTC GT AACMGGATTAGATACCCKG

1193R (reverse primer) PCR primer sequence (each sequence contains different barcode):
Field number (space-delimited), description:
1. Reverse complement of 3’ Illumina adapter
2. i7 index (Listed in Table B.1)
3. Reverse primer pad
4. Reverse primer linker
5. Reverse primer

CAAGCAGAAGACGGCATACGAGAT NNNNNNNN TCATTCCTGG GC ACGTCATCCCCACCTTCC

Read 1 sequencing primer: Read1 799f
Field number (space-delimited), description:
1. Forward primer pad
2. Forward primer linker
3. Forward primer

TACCCCCCTC GT AACMGGATTAGATACCCKG

Read 2 sequencing primer: Read2 1193r
Field number (space-delimited), description:
1. Reverse primer pad
2. Reverse primer linker
3. Reverse primer

TCATTCCTGG GC ACGTCATCCCCACCTTCC

Index 1 sequence primer: IndexRead 1193r
Field number (space-delimited), description:
1. RC of reverse primer
2. RC of reverse primer linker
3. RC of reverse primer pad

GGAAGGTGGGGATGACGT GC CCAGGAATGA

16S rRNA gene amplification
The V5, V6, and V7 regions of the 16S rRNA gene were amplified from each sample using the 799F (5'-AAC MGG ATT AGA TAC CCK G-3') and 1193R (5'ACG TCA TCC CCA CCT TCC-3') primers. These primers minimize amplification from chloroplast DNA and allow for size selection of bacterial amplicons (~450 bp) from mitochondrial amplicons (~900 bp) (5). Primers were designed as in (6) and also contained Illumina MiSeq adapters, and custom pads, linkers, and barcode sequences:

All following pipetting steps were completed using custom liquid handling robotics scripts on the Freedom Evo 200 (Tecan), unless indicated differently. Each PCR was completed in triplicate. The total PCR volume was 25 µL, which contained 1 µL of 10X diluted DNA template, 0.2 µM of each primer, 1X 5PRIME HotMasterMix(5PRIME, Gaithersburg, MD, USA), and 0.8X SBT-PAR additive (5X stock: 750 mM sucrose, 2 mg/mL BSA, 1% Tween-20, 8.5 mM Tris-Cl pH 7.5) (7). PCR amplification consisted of an initial denaturation step of 2 minutes at 94 C, followed by 35 cycles of denaturation at 94 C for 30 s, annealing at 54.3 C for 40 s, elongation at 68 C for 40 s, followed by a final elongation of 7 minutes at 68 C. Replicate reactions were pooled and amplicons purified with an equal volume of Axygen AxyPrep Mag PCR Clean-Up bead solution (Corning, Tewksbury, MA, USA) using the manufacturer's standard protocol. Amplicon concentrations were quantified by fluorimetry (QUANT-iT PicoGreen dsDNA Assay Kit, Life Technologies, Carlsbad, CA, USA), and 30 ng or a maximum of 30 µL per sample were pooled for 576 samples per sequencing run. The length distribution and purity of the final pools were visualized with agarose gels. Primer dimers and mitochondrial amplicons were removed by first concentrating each amplicon pool 20X (Savant SPD121P SpeedVac Concentrator, Thermo Scientific), followed by BluePippin (Sage Science, Beverly, MA, USA) purification for 300-700 bp using the manufacturer's standard protocol.

Sequencing
The final DNA concentrations were determined, and the amplicon pools were sequenced using the Illumina MiSeq platform and MiSeq V2 Reagent Kits (Illumina, San Diego, CA, USA). Runs produced paired-end 250 bp reads (MiSeq Control Software v2.5.0.5) and MiSeq Reporter v2.5.1.3 demultiplexed samples based on dual indices. Four MiSeq runs each pooled six 96-well sample plates. MiSeq software removed reads not paired with an index and reads matching PhiX. The sample plate and MiSeq run numbers were included in a metadata file for the samples along with the sampling site and year, tissue type, developmental stage, and plant identifier.

Data filtering and reduction
Since DADA2 relies on some initial assumptions about the error rate, we first examined the effect of different EE thresholds on the resulting feature table. While lower thresholds generally let more features and samples through, we noticed that even with a loose error threshold (~1/100 bases) there was a group of samples with a very low fraction of reads passing the filter. After pruning the samples to retain only those with 50% or more of reads passing the EE4 filter, 1427 samples remained.

The 1427 sets of forward and reverse reads were loaded into a QIIME2 artifact to tally the amplicon sequence variants present in each sample and perform quality filtering on the count table. Within QIIME2, we used cutadapt to remove primers and ran DADA2 to model errors and find amplicon sequence variants (ASVs). The initial table included 1421 samples with 10,987 sequence variants. We used the taxonomy created with the procedure described below to filter out reads matching mitochondrial or chloroplast sequences (removed 35 sequence variants and 102 samples). We filtered samples with notes in the metadata indicating any irregularities in the collection (removed 45 samples). We then filtered sequence variants with a frequency lower than 2 counts and samples with fewer than 10 reads (removed 19 features and 8 samples). This left a table with 10,803 taxa and 1,272 samples.

To generate the taxonomy we used the QIIME-SILVA bacterial 16S sequence database, release 128. The database taxonomy was constrained to seven levels (domain, phylum, class, order, family, genus, species) and taxonomy was assigned at a given level if on 99% of sequences shared an assignment. The sequences for primers 799F and 1193R were used to extract reads of 16S sequences in the database. The extracted reads were used to build a classifier using QIIME2's naive-bayes method. The classifier was used with the sklearn algorithm in QIIME2 to generate taxonomy assignments for the sequence variants. These taxonomic assignments were used to filter the table and list of sequences for mitochondrial and chloroplast sequences.

To generate a phylogeny for the sequence variants, QIIME2 was used to align the sequences with MAFFT and to infer (fasttree) and root a phylogenetic tree (phylogeny midpoint-root). The tree was imported along with the DADA2-generated ASV count table, the taxonomy, and the metadata into a phyloseq object in R for analysis.

Analysis in R
Sequence data were analyzed in R (version 3.4.4) (8). Count table transformations, pruning, and rarefaction as well as distance matrix calculation and ordination were performed with packages phyloseq (9) and vegan (10). PERMANOVA tests were performed with the adonis function. The VST transformation was performed using the DESeq2 package (11). Phylogenetic analysis was performed with ape and picante (12,13). Figures and supplemental figures were produced with ggplot2, ggpubr, ggrepel, and directlabels (14,15,16,17).

Two approaches were used to asses the relationship of assemblage membership and sample variables. First, the proportion of ASVs shared between samples in random comparisons and comparisons conditioned on sample variables were compared. For each of the 1195 plant samples in the dataset, five other plant samples were randomly drawn without replacement and the mean proportion of ASVs they shared with the focal sample was calculated. Then, the same procedure was repeated with the five samples drawn only from those of the same tissue type, stage, site, or year as the focal sample. Finally, if samples taken from different tissues of the same plant were available, the average proportion of ASVs shared among these samples was calculated. The frequency distributions of these proportions were compared with a one-sided Wilcoxon rank sum test.

The second approach used a permutational multivariate analysis of variance to test for associations between sample variables and assemblage composition. The ASV counts were randomly subsampled without replacement to obtain an even sampling depth of one thousand reads. Assemblage composition was then quantified with the Raup-Crick dissimilarity index, the Bray-Curtis index, or the unweighted UniFrac distance. These distance matrices were ordinated with principle coordinate analysis (PcoA) to examine how samples grouped. The robustness of this grouping to data filters and normalizations was tested by repeating the ordination with count data subject to a VST or TMM transformation, global abundance and prevalence filters for ASV inclusion, or without rarefaction. Variance in the matrix of sample dissimilarities or distances was compared within and between groups for each sample variable and the variables significantly associated with composition ($\alpha = 0.001$) were used to construct a multivariate model for sample variation. In the multivariate model, tissue type was nested within stage because different tissues emerged at specific stages. Stage was nested within year because the stages sampled in each study year varied (Table 1). The terms representing effects from sample processing were nested because the sample processing plates used for DNA extractions and PCRs were divided across four sequencing runs. These nested terms were ordered by their cumulative R^2 values.

To determine the dependence of these associations on the coarseness of taxonomic grouping in the dataset, tests of the multivariate model were repeated with distance matrices calculated from counts of genera, families, orders, classes, and phyla among the plant samples. For each taxonomic level above ASV, the unassigned ASVs were removed before ASVs were grouped into coarser taxa.

ASVs associated with specific tissues or developmental stages were identified were identified using the signassoc function of the indicspecies package (18,19). This function calculated an indicator value index (IndVal) based on the product of two probabilities: (1) the probability that a sample belonged to a habitat given ASV presence and (2) the probability that an ASV was present if a sample was taken from a habitat. For the habitats defined by each variable (six tissues, six developmental stages, two sites, and two years), indices were calculated independently for each ASV. The null hypothesis that no relationship existed between ASVs and conditions was tested by comparing the empirical index with a distribution generated by randomly permuting the ASVpresence-absence count table. A two-tail p-value was used to select ASVs that are significantly more or less frequently observed in sampled belonging to a given condition ($\alpha = 0.01$).

To investigate how consistently the ASVs driving tissue and stage associations in the dataset behaved across sites and years, the maximum prevalence of an ASV within each replication of the experiment was found. If ASVs reached maximum prevalence in the same tissues at each site and in each year they were observed, then they were considered spatially consistent.

Three metrics for assemblage diversity were compared between samples across plant developmental stages. Phylogenetic distance was measured as the summed branch lengths on the 16S phylogenetic tree between ASVs in the sample, with branch lengths weighted by ASV abundance. Shannon-Wiener indices from rarefied samples were calculated as $H' = -\Sigma^{s}_{i=1}(p_i)(\log_2 p_i)$. Evenness was assessed by the distributions of ASV relative abundances in samples with at least one hundred counts.

The diversity of bacterial lineages present was compared within and between root and rosette leaf samples at each developmental stage. For the 698 plant samples with at least one hundred counts and twenty ASVs, a matrix of pairwise dissimilarities (Raup-Crick, Bray-Curtis, or UniFrac) was generated. The dissimilarities for samples within and across groups were selected from the resulting matrix and compared with probability density plots.

References for Supplementary Methods

(1) Perisin M. *The dynamics of bacterial communities associated with Arabidopsis thaliana.* 2016. (Doctoral dissertation, The University of Chicago).

(2) Platt, A., Horton, M., Huang, Y.S., Li, Y., Anastasio, A.E., Mulyati, N.W., Ågren, J., Bossdorf, O., Byers, D., Donohue, K. and Dunning, M., 2010. The scale of population structure in Arabidopsis thaliana. *PLoS genetics*, *6*(2).

(3) Exposito-Alonso, M., Becker, C., Schuenemann, V.J., Reiter, E., Setzer, C., Slovak, R., Brachi, B., Hagmann, J., Grimm, D.G., Chen, J. and Busch, W., 2018. The rate and potential relevance of new mutations in a colonizing plant lineage. *PLoS genetics*, *14*(2), p.e1007155.

(4) Lundberg, D.S., Lebeis, S.L., Paredes, S.H., Yourstone, S., Gehring, J., Malfatti, S., Tremblay, J., Engelbrektson, A., Kunin, V., Del Rio, T.G. and Edgar, R.C. 2012. Defining the core Arabidopsis thaliana root microbiome. *Nature*, *488*(7409), p.86.

(5) Bodenhausen, N., Horton, M. W., & Bergelson, J. 2013. Bacterial communities associated with the leaves and the roots of Arabidopsis thaliana. *PloS one*, *8*(2), e56329.

(6) Kozich, J.J., Westcott, S.L., Baxter, N.T., Highlander, S.K. and Schloss, P.D., 2013. Development of a dual-index sequencing strategy and curation pipeline for analyzing amplicon sequence data on the MiSeq Illumina sequencing platform. *Appl. Environ. Microbiol.*, *79*(17), pp.5112-5120.

(7) Samarakoon, T., Wang, S.Y. and Alford, M.H., 2013. Enhancing PCR amplification of DNA from recalcitrant plant specimens using a trehalose-based additive. *Applications in Plant Sciences*, *1*(1), p.1200236.

(8) R Core Team (2018). R: A language and environment for statistical computing. R Foundation for Statistical Computing, Vienna, Austria. https://www.R-project.org/.

(9) phyloseq: An R package for reproducible interactive analysis and graphics of microbiome census data. Paul J. McMurdie and Susan Holmes (2013) PLoS ONE 8(4):e61217.

(10) Jari Oksanen, F. Guillaume Blanchet, Michael Friendly, Roeland Kindt, Pierre Legendre, Dan McGlinn, Peter R. Minchin, R. B. O'Hara, Gavin L. Simpson, Peter Solymos, M. Henry H. Stevens, Eduard Szoecs and Helene Wagner (2017). vegan: Community Ecology Package. R package version 2.4-5. https://CRAN.R-project.org/package=vegan

(11) Love, M.I., Huber, W., Anders, S. Moderated estimation of fold change and dispersion for RNA-seq data with DESeq2 Genome Biology 15(12):550 (2014).

(12) Paradis, E. and Schliep, K., 2018. ape 5.0: an environment for modern phylogenetics and evolutionary analyses in R. *Bioinformatics*, *35*(3), pp.526-528.

(13) S.W. Kembel, P.D. Cowan, M.R. Helmus, W.K. Cornwell, H. Morlon, D.D. Ackerly, S.P. Blomberg, and C.O. Webb. 2010. Picante: R tools for integrating phylogenies and ecology. Bioinformatics 26:1463-1464.

(14) H. Wickham. ggplot2: Elegant Graphics for Data Analysis. Springer-Verlag New York, 2016.

(15) Alboukadel Kassambara (2018). ggpubr: 'ggplot2' Based Publication Ready Plots. R package version 0.1.8. https://CRAN.R-project.org/package=ggpubr

(16) Kamil Slowikowski (2018). ggrepel: Automatically Position Non-Overlapping Text Labels with 'ggplot2'. R package version 0.8.0. https://CRAN.R-project.org/package=ggrepel

(17) Toby Dylan Hocking (2018). directlabels: Direct Labels for Multicolor Plots. R package version 2018.05.22. https://CRAN.R-project.org/package=directlabels

(18) Cáceres, M. D. (2020). How to use theindicspeciespackage (ver. 1.7.8) https://cran.r-project.org/web/packages/indicspecies/vignettes/indicspeciesTutorial.pdf

(19) Cáceres, M. D., & Legendre, P. (2009). Associations between species and groups of sites: indices and statistical inference. *Ecology*, *90*(12), 3566-3574.
