# Supplementary material for: Natural Bacterial Assemblages in Arabidopsis thaliana Tissues Become More Distinguishable and Diverse during Host Development
Source: mBio. 2021 Jan 19;12(1):e02723-20. doi: 10.1128/mBio.02723-20 (PMC7845642; doi:10.1128/mBio.02723-20)
Supplement: TEXT S2 [file mBio.02723-20-s0002.pdf]

Plants hosted bacterial assemblages distinct from those in the surrounding soil.

Assemblages in plant tissues were depleted of phylum Acidobacteria and the classes Thermoleophilia, Bacilli, and Gemmatimonadetes relative to the surrounding soil. Data from the second study year, during which both plants and soil were collected at each timepoint, were filtered to exclude rare ASVs (total counts less than one thousand) and the relative abundance of each ASV was calculated for both plant root and soil samples. In each class, the distributions of these variant relative abundances were compared between soil and plant samples with a Kruskal-Wallis rank test ($\alpha = 0.001$) and the mean relative abundances in soil and plant samples were compared with a 95% confidence interval (Figure 1A). The relative abundance distributions of variants in Acidobacteria, Acidobacteria subgroup 2, Thermoleophilia, Bacilli, and Gemmatimonadetes differed significantly between soil and plant samples. The mean relative abundances of variants in these classes were reduced in roots compared to soil, indicating that the host plant might select against them during colonization.

Assemblages in plant tissues were mostly composed of class Actinobacteria and the subphyla α, β, and γ of Proteobacteria. To examine which clades were most abundant in plant assemblages, raw sequence counts for ASVs were summed for all plant samples and the mean abundance for variants in each class was compared with a 95% confidence interval (Figure 1B). Variants of Actinobacteria, Alphaproteobacteria, Betaproteobacteria, and Gammaproteobacteria on average had higher counts than variants of other classes by roughly one thousand, indicating that members of these lineages were the most common colonists of plant tissues.

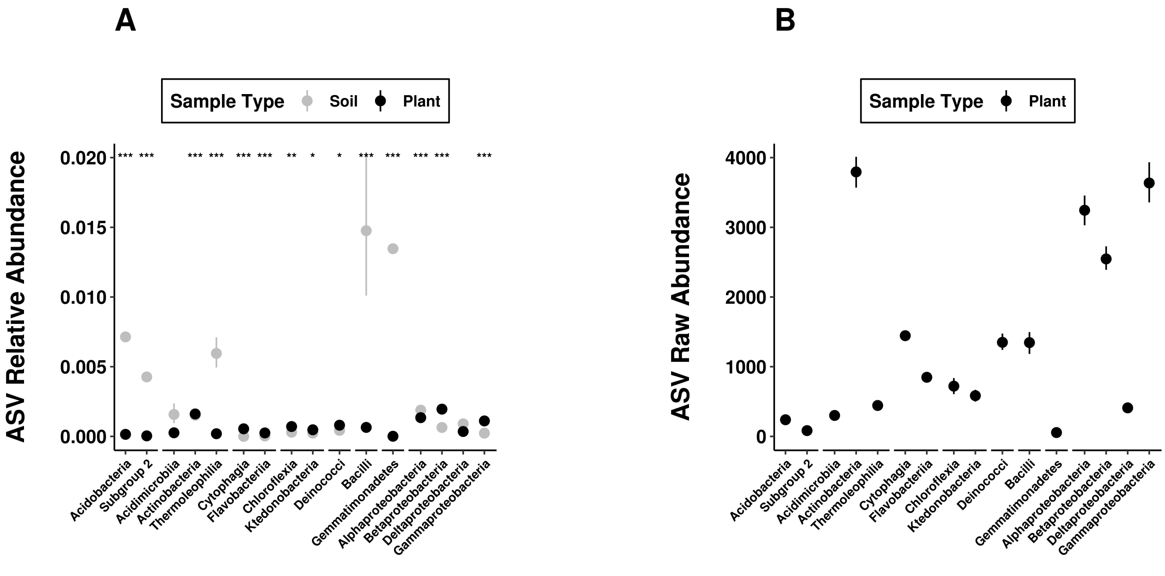

Plant assemblages were distinct from the surrounding soil and composed mostly of variants in class Actinobacteria and Proteobacteria subphyla α, β, and γ. These plots include observations of the 680 ASVs in the dataset with more than 1000 total counts among 1181 plant and 77 soil samples. (A) The relative abundance of ASVs was calculated in both root samples and soil samples from all timepoints, sites, and years. The mean relative abundance for variants in each class, grouped by phylum on the x-axis, is plotted with a bootstrapped 95% confidence interval for both soil (gray) and plant (black) samples. A Kruskal-Wallis test indicates significant differences between soil and plant samples in the relative abundance distributions for variants of a class at alpha < 0.0001 (***), 0.001 (**), and 0.01 (*). (B) For plant samples, the counts of ASVs across tissues, stages, sites, and years were summed. The mean abundance for variants in each class, grouped by phylum on the x-axis, is plotted with a bootstrapped 95% confidence interval.
