# Supplementary material for: Natural Bacterial Assemblages in Arabidopsis thaliana Tissues Become More Distinguishable and Diverse during Host Development
Source: mBio. 2021 Jan 19;12(1):e02723-20. doi: 10.1128/mBio.02723-20 (PMC7845642; doi:10.1128/mBio.02723-20)
Supplement: TABLE S3 [file mBio.02723-20-st003.pdf]

TABLES S3 Variables tested for association with dissimilarity index matrices by PERMANOVA

			Raup-Crick index				Bray-Curtis dissimilarity			UniFrac distance		
Variable	Description	Values	DOF	F	R^2	Pr (>F)	F	R^2	Pr (>F)	F	R^2	Pr (>F)
Tissue	The part of the plant washed, ground, and sampled for DNA extraction	Roots, Rosettes, Stems, Cauline Leaves, Flowers, Siliques	5	577.184	0.803	< 0.001	23.909	0.145	< 0.001	21.600	0.133	< 0.001
Stage	The developmental stage of the plant when the tissue was harvested	Two Leaf, Four Leaf, Six Leaf, Eight Leaf, Flowering, Senescent	5	120.622	0.461	< 0.001	14.606	0.094	< 0.001	9.091	0.060	< 0.001
Site	The location of the field where the plant grew	Michigan Extension, Warren Woods	1	202.328	0.222	< 0.001	38.890	0.052	< 0.001	22.897	0.031	< 0.001
Year	The year in which the plant was harvested	1, 2	1	93.356	0.116	< 0.001	21.224	0.029	< 0.001	11.904	0.016	< 0.001
Ecotype	The *A. thaliana* genotype of the plant sampled	BRR4, LI-WP-041, L-R-10, MNF-Che-47, Pent-7, PT1.85, SLSP-69	6	1.717	0.014	0.342	0.931	0.008	0.748	0.802	0.007	0.958
Sample Plate	The 96-well microplate in which sample extraction and 16S amplification were performed	Plate 1 – 24	23	0	0	0.914	1.311	0.042	< 0.001	1.640	0.052	< 0.001
MiSeq Run	The batch in which samples were sequenced on the Illumina MiSeq platform	Run 1 – 4	3	0	0	0.710	2.296	0.010	< 0.001	4.200	0.017	< 0.001
Plant ID	The plant individual from which tissues were harvested	Plant 1 – 376	375	1.253	0.448	0.012	1.172	0.431	< 0.001	1.067	0.408	0.003
