# Supplementary figures and images for: Natural Bacterial Assemblages in Arabidopsis thaliana Tissues Become More Distinguishable and Diverse during Host Development
Source: mBio. 2021 Jan 19;12(1):e02723-20. doi: 10.1128/mBio.02723-20 (PMC7845642; doi:10.1128/mBio.02723-20)

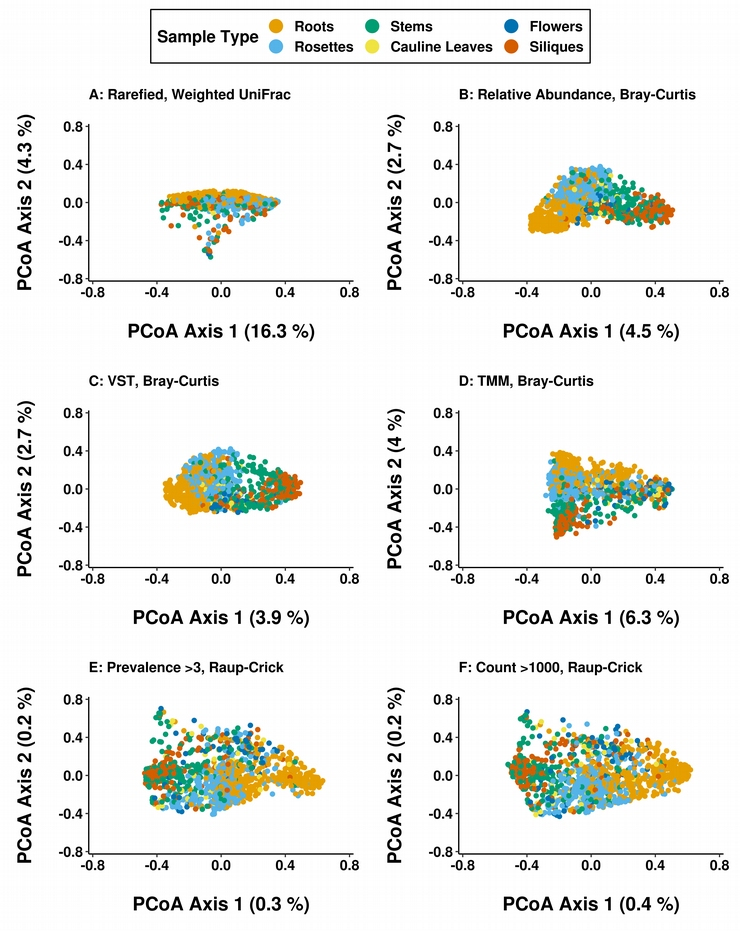

Supplement: FIG S1 [file mBio.02723-20-sf001.tif]
