# Supplementary material for: Natural Bacterial Assemblages in Arabidopsis thaliana Tissues Become More Distinguishable and Diverse during Host Development
Source: mBio. 2021 Jan 19;12(1):e02723-20. doi: 10.1128/mBio.02723-20 (PMC7845642; doi:10.1128/mBio.02723-20)
Supplement: TABLE S4 [file mBio.02723-20-st004.pdf]

TABLE S4 PERMANOVA results for flowering phyllosphere samples

		Raup-Crick index		
	DOF	F	R^2	Pr (>F)
Year : **Tissue**	7	11.385	0.380	0.01
**Site**	1	42.098	0.201	< 0.001
**Year**	1	1.843	0.009	0.421
MiSeq Run : **Sample Plate**	20	0	0	0.917
**MiSeq Run**	3	0	0	0.773
Residuals			0.015	
